# Evaluation of the Airway Space After Distalization in Adults Using the Carrière Motion Appliance: A Cone-Beam Computed Tomography (CBCT) Study

**DOI:** 10.7759/cureus.78184

**Published:** 2025-01-29

**Authors:** Heba Elghawy, Abbadi A El Kady, Walaa Elgemeay

**Affiliations:** 1 Orthodontics, Suez Canal University, Ismailia, EGY; 2 Orthodontics, Faculty of Dentistry, Suez Canal University, Ismailia, EGY

**Keywords:** airway volume, carriere motion ii, cbct, class ii malocclusion, distalization

## Abstract

Background: Distalization can be done with either intraoral (IO) or extraoral (EO) appliances and is one of the most prevalent methods for correcting class II molars. In recent decades, it has become more pervasive to treat class II malocclusions without taking teeth out. Persons with a neutro-basal jaw base relationship should distalize their teeth to make the dental arch wider in order to prevent the need to remove permanent teeth. The purpose of this investigation was to apply cone-beam computed tomography (CBCT) to examine how airway space changes in response to varying degrees of distalization.

Methods: Eighteen adult patients between the ages of 18 and 30 with complete dentition were instructed to wear the Carrière Motion Appliance (CMA) for four to six months. CBCT scans were obtained both before and after distalization for further evaluation. Pre- and post-distalization CBCT pictures were obtained for evaluation.

Results: This study's findings clearly show that Carrière Motion II improves airway volume (AWV) and minimum constricted area (MCA) to a modest degree. The TAV (total airway volume) increased significantly by 12.5% (from 39.9 ± 2.7 mL in pre-distalization to 44.9 ± 2.6 mL in post-distalization). Furthermore, the MCA increased significantly (at *P* ≤ 0.0001) by 62.7% between pre- and post-distalization. Airway volume measures of the nasopharyngeal, oropharyngeal, and hypopharyngeal levels also showed significant increments. Specifically, the nasopharyngeal, hypopharyngeal, and oropharyngeal airway volumes increased by 5.7%, 78.7%, and 15.6%, respectively.

Conclusions: The hypopharyngeal and oropharyngeal airways exhibit the largest volume gain compared to the nasopharyngeal airways. The upper TAV and MCA were clearly increased by the CMA.

## Introduction

Many approaches could be used for the treatment of a class II malocclusion. One of the traditional approaches in orthodontics to treat class II molars and gain space is called distalization. This can be done with either intraoral appliances (IOAs) or extraoral appliances (EOAs). The non-extraction approach to treating class II malocclusions is currently acquiring a great deal of popularity. In some cases, patients with skeletal class I along with other indications of distalization can have the benefit of distalizing permanent teeth without the need for extraction in order to achieve sagittal expansion of the dental arch [1].

By gaining a greater grasp of tooth movement, bone physiology, biomechanics, and more recent biomaterials, sophisticated distalization procedures have been vastly improved throughout the years. Clinicians recommend IOAs than EOAs because they facilitate distal molar movement better [2].

Extraoral anchoring's key limitations are that it requires patient assent and that it lacks visual appeal as a treatment option. Intraoral procedures like pendulum appliances, magnets, distal jet appliances, nickel-titanium open coil springs, and a number of others were utilized in order to distalize molars [3]. This was done in order to get beyond the limitations that were placed on the procedure.

In 2004, a modern appliance was set up by Luis Carrière, bearing his name, known as the Carrière Motion appliance (CMA). The CMA is a piece of technology that addresses the patient's sagittal dimension to create a class I platform before full orthodontic treatment [4]. This is done by addressing the patient's sagittal dimension.

The CMA is used to remedy cases to a class I platform for the macro-adjustment of the occlusion during the initial stage of therapy when patient compliance is at its optimum. This is the phase in which the occlusion is most easily treated. After that, the dentist has the option of finishing the case with either a fixed appliance technique or an aligner appliance system [4].

Since the angle defined a class I occlusion based on the presence of a molar connection, the Carriѐre technique subscribes to a more inclusive definition of class I called the class I platform. This definition states that a class I occlusion must have perfect buccal segment intercuspation from cuspids to molars and that centric relation coincides with centric occlusion. When the maxillary and mandibular cusps come together adequately in a class I relationship, the buccal segment may spontaneously settle itself into a class I connection if the maxillary first molars are appropriately distally rotated to the back. This occurs when the buccal segment is properly aligned [5].

This technique is analogous to initially correcting a class II occlusion with a functional appliance, followed by a phase of fixed appliance treatment. The procedure may also involve a combination of the two approaches [6]. The appliance is made up principally of an upper sectional and a bonded bracket that is placed on the lower molars. In addition, there are a variety of methods that are used to improve lower anchoring. The design allows us to use class II elastics [7].

Lately, airway examination and the influence of various treatment techniques on the airway space have garnered considerable interest from scientists, and contradictory results have been reported in the medical literature. Extraction therapy has been demonstrated in multiple investigations to result in a reduction in the size of the pharyngeal airway. It has been claimed that extraction treatment may increase the risk of obstructive sleep apnea (OSA) in individuals [8]. Eventually, a recent study confirmed that orthodontic retraction with premolar extractions neither exhibits a significant reduction in airway dimensions nor increases the risk of OSA [9].

However, the effect of distalization on airway volume in class II patients using the CMA has not yet been adequately evaluated. The goal of this study was to examine with the help of CBCT (cone-beam computed tomography), the connection that exists between the degree of distalization and the alterations that take place in the airway space.

Three-dimensional (3D) imaging has evolved greatly in the last two decades and has found applications in orthodontics, as well as in oral and maxillofacial surgery. In 3D medical imaging, a set of anatomical data is collected using diagnostic imaging equipment, processed by a computer, and then displayed on a 2D monitor to give the illusion of depth. Depth perception causes the image to appear in 3D. Hence, in this study, CBCT imaging techniques were used to give more accurate results regarding pre- and post-distalization treatment in terms of airway space measurements [10].

## Materials and methods

Patients

Sample Size Calculation

The current study was conducted on 18 adult patients with the permission of the Faculty of Dentistry, Suez Canal University's Research Ethics Committee. To assess and evaluate changes in airway space after distalization using the CMA in adult patients, a paired-sample t-test (dependent samples t-test) or other equivalent test for nonparametric data was presented. A total sample size of 18 was required to detect an effect size of 0.65 with a power of 80% (1-β = 0.80) and a two-tailed significance level of 5% (p-value ≤ 0.05). In accordance with previous estimations, a total sample size of 18 with a power of 0.80 (80%) was in use (the motion 3D class II appliance, Carriѐre Motion, Henry Schein Orthodontics, USA). G*Power software version 3.1.9.2 (Universitat Kiel, Germany) was employed to determine the sample size [11].

Clinical Trial Registration

The study protocol was registered on clinicaltrial.gov with registration no. NCT05261217.

Participants

Patients were selected from the outpatient clinic at Suez Canal University (SCU)'s Department of Orthodontics, Faculty of Oral and Dental Medicine. The Faculty of Oral and Dental Medicine's Research Ethical Committee at SCU approved this study with approval number 192/2019. All patients were informed about the procedures and shown animated illustration videos.

Inclusion and Exclusion Criteria

The patients who were chosen had met the following inclusion criteria: 1) adults aged 18 to 30 with full permanent dentition; 2) dental class II malocclusion ranging from a half unit to a full-unit class II relationship bilaterally and where the treatment plan did not include upper arch extraction; 3) a normal skeletal maxilla with a normal or mild to moderate retrognathic mandible; 4) a normal or horizontal growth pattern; 5) to provide a reasonable prognosis, all patients should have good oral hygiene and follow motivational instructions; 6) the absence of erupted maxillary third molars; 7) the absence of any systemic disease or health problems; and 8) no oral breathing problems reported by the patients.

Preoperative intervention

Medical History Questionnaire

The patients filled out a medical questionnaire to rule out any potential systemic diseases (see Appendix). Furthermore, the patients were examined by ear, nose, and throat specialists to ensure that no airway disorders existed.

Dental Case History and Clinical Examination

Each patient provided a detailed dental case history, and all were clinically diagnosed and subjected to extraoral and intraoral examinations. The patients were examined to make sure they met the abovementioned inclusion criteria. Patients who met the prerequisites were referred to periodontal department clinics for further oral hygiene improvement, restorative department clinics for any requisite fillings and the oral and maxillofacial surgery department for extraction of the third maxillary molars if they were fully erupted in the oral cavity.

Patient Records

**Figure 1:**
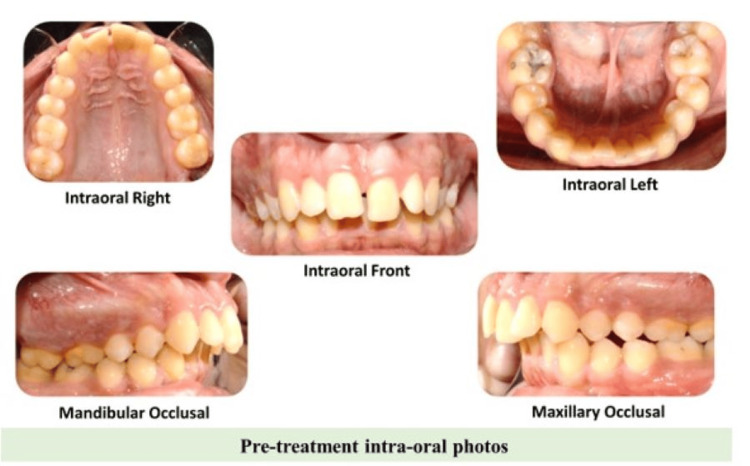
Pre-treatment intra-oral photos

Before and after the treatment period, the following records were obtained from each patient. A total of four extraoral and five intraoral photographs were taken for each patient (Figure 1).

Orthodontic study models

Upper and lower impressions were taken on all patients using alginate impression material (Tropicalgin, Zhermack, Germany). A bite of wax wafer (modeling wax; Cavex, Haarlem, Netherlands) was also acquired. The impressions were made immediately using extra-hard stone, and the model bases were shaped and trimmed to meet orthodontic standards.

CBCT

The patients were subjected to two CBCT scans, one before treatment and one after distalization with the CMA. The CBCT images were captured using a Soredex SCANORA 3D in the radiology department of SCU's Faculty of Dentistry. The same operator performs CBCT scanning using the manufacturer's protocol. The CBCT parameters were set in accordance with the manufacturer's recommendations.

Preparation of the mandibular arch

In this study, the lingual arch was chosen for its simplicity, ease of fabrication, high patient acceptance, and minimal patient care requirement. All patients received a passive lingual holding arch to anchor the lower arch and maintain class II elastics. SCU's Orthodontic Dental Laboratory, which is part of the Faculty of Dentistry, fabricated the device. To make the appliance, the following steps were taken: 1) First, a separation technique was done between the first and second mandibular molars, and 2) lower bands (Ormco, California, USA) were then tried in the patients' mouths to determine the correct and precise sizes for each patient. The size of the bands was carefully selected to fit snuggly around the molars, and a dental impression with alginate impression material (Henry Schein, New York, USA) was taken with the bands fitted in place; the impression was then sent to the laboratory for fabrication of the passive lingual holding arch. 4) Finally, the appliance was precisely cemented in the patients' mouths using glass ionomer cement (Promedica Medicem, Germany).

Preparation of the maxillary arch

The appliance was delivered to all patients bilaterally in the following steps: First, the effective size was taken using a CMA Ruler provided with the appliance. Then, a measurement was taken from the midpoint on the buccal surface of the maxillary first molar to the midpoint on the labial surface of the maxillary canine. After that, the traditional bonding technique was applied on the labial and buccal surfaces of maxillary canines and maxillary first molars. Finally, activation by using class II elastics (Wildlife Series™ Elastomerics, American Orthodontics, Washington, USA) was applied from the selected source of anchorage to the hook provided on the maxillary anterior pad of the CMA (Carriѐre Motion II, Henry Schein Orthodontics, USA).

Patients’ orientation

All patients were positioned upright on the CBCT unit's chair in the natural head position, with their teeth at maximum intercuspation and their lips and tongue at rest. The landmarks and reference planes were identified using the On-Demand application software (CyberMed, Seoul, Republic of Korea) after the DICOM files from the CBCT scans were opened. The 3D On-Demand application software tool's threshold for controlling the filling degree of the air was set to -1000; below this level, the software detected no air.

After reorienting the photo, the landmarks were located on the 3D volume, and the generated multiplanar slice locator in the three cuts was refined (axial, sagittal, and coronal). The readings were generated using the prerecorded special analysis. All measurements were taken by the same observer and were replicated two weeks later. The intra-observer reliability was reduced because mean values were used.

Airway volume analysis using the 3D On-Demand program

**Figure 2:**
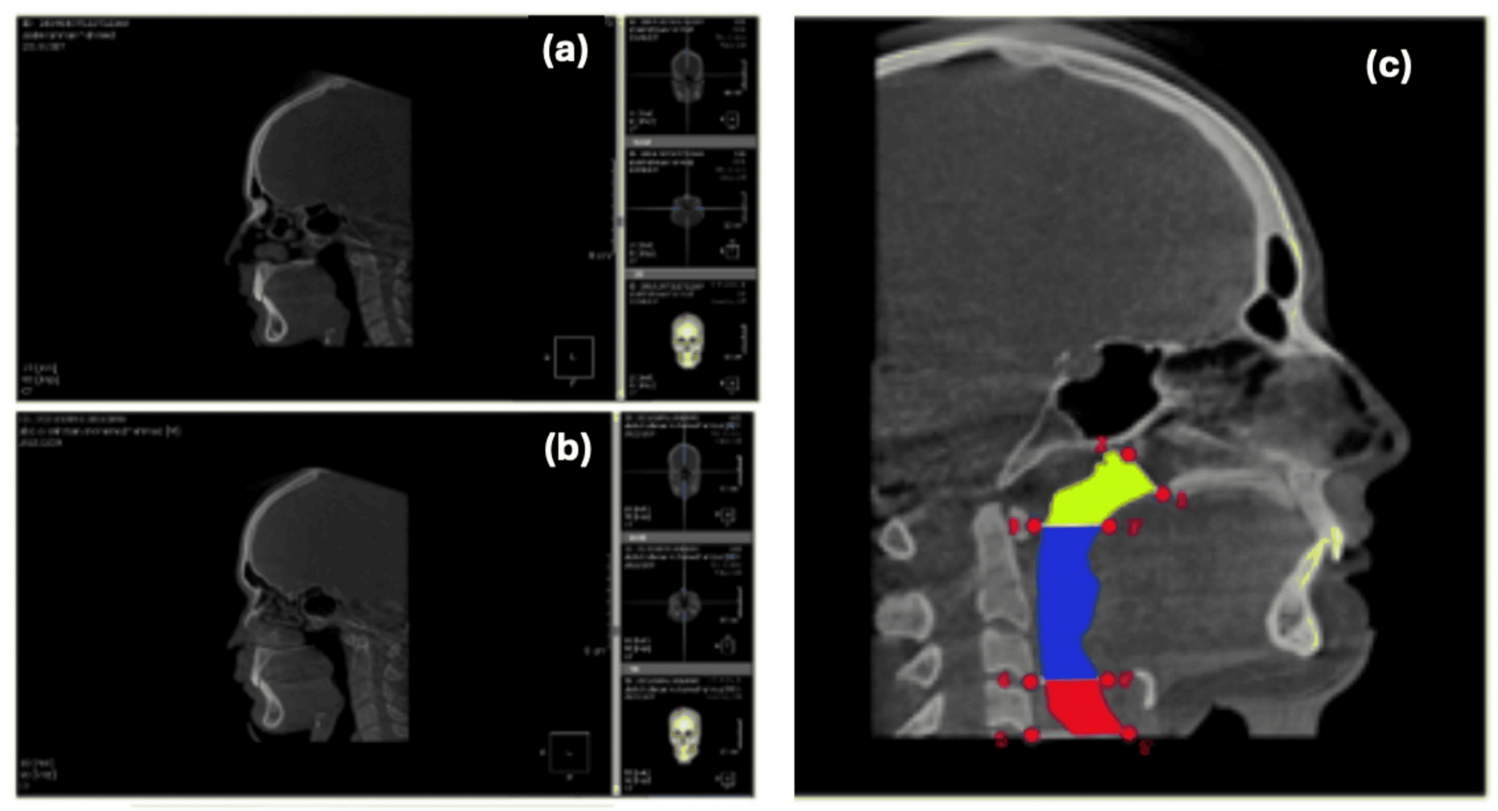
A sagittal view with localized landmarks to measure volume of the nasopharynx in both pre-distalization (a) and post-distalization (b). A diagram showing landmarks, reference planes, and airway zones (c). Landmarks: PNS (most posterior point on the bony hard palate; PTM, intersection of the inferior border of the foramen rotundum with the posterior wall of the pterygomaxillary fissure); C1 (inferior anterior point of Atlus); C3 (inferior anterior point of the third cervical vertebra); C4 (inferior anterior point of the fourth cervical vertebra). Reference planes: (1-2) PNS-PTM plane; (3-3') C1 plane; (4-4') C3 plane; (5-5') C4 plane. Airway zones: yellow (nasopharyngeal airway); blue (oropharyngeal airway); red (hypopharyngeal airway)

Airway volume analysis was carried out in the following manner: 1) The selected portion's borders were drawn based on the identified landmarks. 2) The coronal, axial, and sagittal views were adjusted as follows: a) The axial view was adjusted until the PNS was visible, and b) the coronal and sagittal views were adjusted for standardization. To select the desired area, the Overlay (VOI) icon was utilized. The landmarks (Table 1) were located to create the rectangle’s sides for standardization on the maximized sagittal view, which are the anterior border at the PNS perpendicular to Frankfort, posterior border at C1 perpendicular to Frankfort, inferior border at C1 parallel to Frankfort, and superior joining anterior and posterior border. The nasopharyngeal, oropharyngeal, and hypopharyngeal airways were all measured using landmarks specific to each airway segment. The software determines the narrowest cross-section area of the airway to be measured automatically (Figure 2).

**Table 1:** Landmarks and reference planes

Landmarks and reference planes (abbreviation)	Definition
Landmarks:	
1. Posterior nasal spine (PNS)	1. Most posterior point on the bony hard palate
2. Pterygomaxillary points (PTM)	2. Intersection of the inferior border of the foramen rotundum with the posterior wall of the pterygomaxillary fissure
3.C1	3. Inferior anterior point of Atlus
4. C3	4. Inferior-anterior point of the third cervical vertebra
Reference planes	
1. PNS plane	1. Plane passing through PNS perpendicular to the sagittal plane
2. PTM-PNS	2. Plane connecting the right and left PTM passing PNS
3. C3 plane	3. Plane passing through the inferior anterior point of the third cervical vertebra perpendicular to the sagittal plane
4. Frankfort horizontal (FH)	4. Plane connecting the most inferior border of orbital to the most superior border of the porion
5. C1 plane	5. Plane passing through the inferior-anterior point of the atlas perpendicular to the sagittal plane
6. C3 plane	6. Plane passing through the inferior-anterior point of the third cervical vertebra perpendicular to the sagittal plane

Statistical analyses

IBM SPSS Statistics for OS, Version 29.0 (IBM Corp., Armonk, NY) was used for statistical analysis. Data are represented using the mean and standard deviation. Cronbach's alpha and interclass correlation were utilized to examine intra-observer reliability. To compare pre-distalization and post-distalization, the paired t-test was employed [12].

## Results

The findings of the present study include data from nasopharyngeal, oropharyngeal, hypopharyngeal, and total airway spaces.

Nasopharyngeal airway space

The intra-observer difference in the nasopharyngeal airway space is shown in Table 2. Airway space measures in terms of nasopharyngeal were measured by two separate readings (R1, R2) of one observer. Interclass correlation (ICC) at the 0.05 level was used to evaluate the levels of intra-observer reliability and internal consistency, respectively. As a consequence of this, excellent intra-observer reliability was reported between readings 1 and 2 in pre-distalization (ICC = 0.980) and between readings in post-distalization (ICC = 0.965).

**Table 2:** Nasopharyngeal airway space in readings 1 and 2 and both pre-distalization and post-distalization. Intra-observer reliability and internal consistency were evaluated by interclass correlation (ICC) at p ≤ 0.05 ICC: interclass correlation; sign.: significance

Time of observation	Nasopharyngeal airway space (mL; Mean ± SD)			Intra-observer reliability	
	Reading 1	Reading 2	Total	ICC	Sign.
Pre-distalization	13.18 ± 5.07	13.08 ± 4.84	13.13 ± 4.95	0.980	<0.001***
Post-distalization	14.06 ± 4.91	13.69 ± 4.70	13.88 ± 4.80	0.965	<0.001***

In reading 1, the nasopharyngeal airway space varied from 7.39 to 26.20 ml, with an average value of 13.18 ± 5.07 mL. In reading 2, the range was from 6.30 to 25.53 mL, with an average value of 13.08 ± 4.84 mL. However, in readings 1 and 2, the nasopharyngeal airway space ranged between 8.10 and 26.20 mL and from 8.36 to 27.05 mL, with an average (SD) of 14.06 ± 4.91 mL and 13.69 ± 4.70 mL, respectively. After distalization, the range was 8.10 to 27.05 mL (Table 3).

**Table 3:** Nasopharyngeal airway space in readings 1 and 2 and both pre-distalization and post-distalization. Intra-observer reliability and internal consistency were evaluated by the interclass correlation (ICC) at p ≤ 0.05 ICC, interclass correlation; sign., significance

Time of observation	Nasopharyngeal airway space (mL; mean ± SD)			Intra-observer reliability	
	Reading 1	Reading 2	Total	ICC	Sign.
Pre-distalization	13.18 ± 5.07	13.08 ± 4.84	13.13 ± 4.95	0.980	<0.001***
Post-distalization	14.06 ± 4.91	13.69 ± 4.70	13.88 ± 4.80	0.965	<0.001***

**Figure 3:**
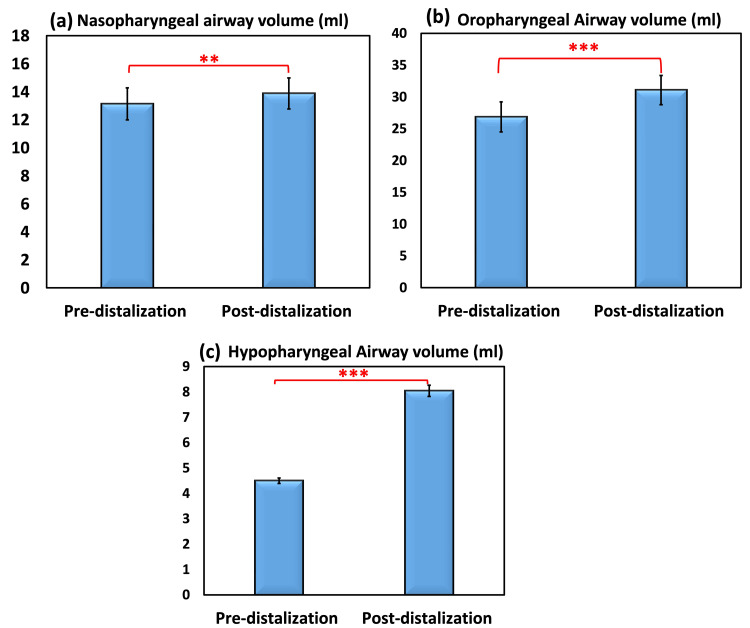
Nasopharyngeal (a), oropharyngeal (b), and hypopharyngeal (c) airway space volumes (mL) in both pre-distalization and post-distalization. A paired samples t-test with a p-value ≤0.05 was used to compare the pre- and post-test results.

The minimum, maximum, mean, standard error, and standard deviation were reported as descriptive statistics of the nasopharyngeal airway space volume (mL) in Figure 3a. Before distalization, the nasopharyngeal airway space volume (mL) ranged from 6.30 to 26.20, with an average SE of 13.13 ± 1.15 mL. On the other hand, the nasopharyngeal airway space after distalization varied from 8.10 to 27.05 mL, with a mean and SD of 13.88 and 1.12 mL, respectively. The difference in nasopharyngeal space before and after distalization was measured to be 0.74 mL, which corresponds to a change percentage of 5.67%. The results of the paired samples t-test confirmed that there was a significant difference in the volume of the nasopharyngeal airway gap before and after distalization. According to the Pearson correlation coefficient, the connection between pre- and post-distalization in both readings was a strong direct very significant correlation (0.978). This was revealed by the coefficient.

Oropharyngeal airway space

The oropharyngeal airway space volume (mL) was determined by taking two separate readings (R1, R2) from the same observer. The mean and standard deviation are shown in Table 4 and Figure 3b, respectively, to illustrate the intra-observer difference in oropharyngeal airway space volume. The ICC at the 0.05 level was used to evaluate the levels of intra-observer reliability and internal consistency, respectively. As a consequence of this, a high outstanding intra-observer reliability was obtained between readings 1 and 2 in pre-distalization (ICC = 0.985) and between readings in post-distalization (ICC = 0.995).

**Table 4:** Oropharyngeal airway space in readings 1 and 2 and both pre-distalization and post-distalization. Intra-observer reliability and internal consistency were evaluated by the interclass correlation (ICC) at p ≤ 0.05. ICC, interclass correlation

Time of observation	Oropharyngeal airway space (mL; mean ± SD)		Intra-observer reliability	
	Reading 1	Reading 2	ICC	Sign.
Pre-distalization	26.77 ± 10.02	26.95 ± 10.29	0.985	<0.001***
Post-distalization	30.74 ± 9.75	31.42 ± 10.17	0.995	<0.001***
Paired t-test	p<0.001***			

In reading 1, the oropharyngeal airway space before distalization ranged from 10.08 to 43.55 mL, with an average of 26.77±10.02 mL. In reading 2, the oropharyngeal airway space before distalization ranged from 9.67 to 44.80 mL, with an average of 26.95±10.29 mL. By contrast, the oropharyngeal airway space volume after distalization ranged from 13.03 to 47.16 mL in reading 1 and from 13.55 to 49.92 mL in reading 2, with an average (SD) of 30.74±9.75 mL and 31.42±10.17 mL; respectively (Table 4, Figure 3b).

The minimum, maximum, mean, standard error, and standard deviation of the oropharyngeal airway space volume (mL) are provided in Table 4 and Figure 3b, respectively. These are the descriptive statistics of the volume.

The oropharyngeal airway space volume (mL) before distalization ranged from 9.67 to 44.80 mL, with a mean SE of 26.86 mL and a standard error of 2.36 mL. However, the oropharyngeal airway space volume (mL) after distalization ranged anywhere from 13.03 to 49.92, with a mean value of 31.08 mL and a standard error of 2.32 mL. Before and after distalization, the amount of space in the oropharynx changed by 4.22 mL, which corresponds to a change percentage of 15.73%. According to the results of the paired samples t-test, there was a difference between pre- and post-distalization that was extremely significant (p ≤ 0.001***). The association between pre-distalization and post-distalization was a strong direct significant link (r = 0.972) based on the Pearson correlation coefficient.

Hypopharyngeal airway space volume

The hypopharyngeal airway space volume (mL) was determined by taking two separate readings (R1, R2) from the same observer. The mean and standard deviation are shown in Table 3 and Figure 3c, respectively, to illustrate the intra-observer difference in the hypopharyngeal airway space volume. The ICC at the 0.05 level was used to evaluate the levels of intra-observer reliability and internal consistency, respectively. Because of this, a high outstanding intra-observer reliability was observed between R1 and R2 in pre-distalization (ICC = 0.845; sign. at P ≤ 0.001***), as well as between readings in post-distalization (ICC = 0.948; sign. at P ≤ 0.001***).

In pre-distalization readings, the range of the hypopharyngeal airway space was between 3.90 and 5.42 mL, with an average of 4.49 ± 0.11 mL. In reading 2, the range of the hypopharyngeal airway space was between 3.76 and 6.02 mL, with an average of 4.51 ± 0.15 mL. By contrast, the volume of the hypopharyngeal airway space after distalization ranged from 4.96 to 9.00 mL in reading 1 and from 5.07 to 9.82 mL in reading 2, with an average SD of 4.50 ± 0.54 mL and 8.04 ± 0.98 mL, respectively (Table 5, Figure 3c).

**Table 5:** Hypopharyngeal total airway volume in readings 1 and 2 and both pre-distalization and post-distalization. Intra-observer reliability and internal consistency were assessed by interclass correlation (ICC) at p ≤ 0.05. ICC, interclass correlation

Pre/post	Total airway volume (ml)			Inter-observer reliability		
	Reading 1	Reading 2	Total		ICC	Significance
	Mean ± SD	Mean ± SD				
Pre-distalization	39.94 ± 12.03	40.03 ± 11.45	39.99 ± 11.74		0.985	<0.001***
Post-distalization	44.80 ± 11.28	45.11 ± 11.39	44.96 ± 11.34		0.990	<0.001***

The minimum, maximum, mean, standard error, and standard deviation of the hypopharyngeal airway space volume (mL) are provided in Table 3 and Figure 3c, respectively. These are the descriptive statistics. Before distalization, the hypopharyngeal airway space volume (mL) was found to range anywhere from 3.76 to 6.02, with a mean SE of 4.50 ± 0.128 mL of the standard error. However, after the distalization procedure, the hypopharyngeal airway space was anywhere from 4.96 to 9.82 mL, with an average SD volume of 8.04 mL. The difference in the amount of space in the hypopharynx between before and after distalization was 3.54 mL, which corresponds to a change percentage of 78.81%. The results of the paired samples t-test demonstrated that there was a statistically significant difference in the volume of the hypopharyngeal airway gap before and after distalization. According to the Pearson correlation coefficient, there was a strong direct highly significant connection (0.529; sign. at p ≤ 0.001***) between pre-distalization and post-distalization in both readings. This correlation was proven to be strong.

Total airway space

The intra-observer difference in the total airway space volume (nasopharyngeal + oropharyngeal + hypopharyngeal) is shown in Table 4. The total airway space volume was measured in milliliters and was determined by two separate readings taken by the same observer (R1, R2). The ICC at the 0.05 level was used to evaluate the levels of intra-observer reliability and internal consistency, respectively. In light of this, a high outstanding intra-observer reliability was observed between R1 and R2 in pre-distalization (ICC = 0.981; sign. at p ≤ 0.001***), as well as between readings in post-distalization (ICC = 0.986; sign. at p ≤ 0.001***). Reading 1 showed that the total airway space could be anywhere from 24.58 to 64.35 mL, with an average of 44.43 ± 11.95 mL. Reading 2 showed that the total airway space could be anywhere from 23.15 to 61.56 mL, with an average of 44.54 ± 11.39 mL. By contrast, the total airway space volume after distalization ranged from 31.08 to 68.45 mL and from 32.44 to 69.79 mL in readings 1 and 2, respectively, with an average SD of 52.73 ± 10.97 mL and 53.27 ± 11.09 mL for readings 1 and 2, respectively (Table 6). 

**Table 6:** Total airway space in readings 1 and 2 and both pre-distalization and post-distalization the interclass correlation (ICC) at p ≤ 0.05 were used to measure the reliability and consistency within each observer. ICC, interclass correlation; r, Pearson's correlation coefficient

Pre/post	Total airway volume (ml)			Inter-observer reliability		
	Reading 1	Reading 2	Total		ICC	Significance
	Mean ± SD	Mean ± SD				
Pre-distalization	44.43 ± 11.95	44.54 ± 11.39	44.48 ± 11.67		0.981	<0.001***
Post-distalization	52.73 ± 10.97	53.27 ± 11.09	53.00 ± 11.03		0.986	<0.001***
Paired t-test signifiance	p ≤0.001***					
Correlation (r)	r = 0.989					

The minimum, maximum, mean, standard error, and standard deviation were reported as descriptive statistics of the total airway space volume (mL). Table 4 comprised of the following headings: The total airway space volume (mL) before distalization ranged from 23.15 to 64.35 mL, with a mean SD of 44.48 ± 11.67 mL. However, the total airway space volume (mL) after distalization ranged from 31.08 to 69.79 mL, with an average of 53.00 ± 11.03 mL. Between before and after the procedure, there was a change of 8.51 mL in the total airway space, which corresponds to a change percentage of 19.14%. The results of the paired samples t-test demonstrated that there was a difference in the total airway space volume between before and after distalization that was statistically significant (p ≤ 0.001***). According to the Pearson correlation coefficient, the connection in the total airway space between pre-distalization and post-distalization was a highly direct significant link (r = 0.989).

## Discussion

Class II malocclusion is characterized by the presence of a mandibular anteroposterior deficiency in comparison to the maxilla, an enlarged maxilla prominence when compared to the mandible, or a mix of both skeletal and dental features. According to research that has been done so far, there is a link between class II malocclusion and a smaller pharyngeal airway.

The first attempt was to associate the pharyngeal airway with various anteroposterior malocclusions. This was supported by Ceylan and Oktay [13], who continued the line of research. As a result, the experts concluded that the nasopharyngeal areas and depths of the participants with class II malocclusion were much lower than those of the subjects with normal occlusion. This was due primarily to these individuals' ability to maintain mandibular posture, which puts them at risk of developing obstructive sleep apnea [14].

It has been undertaken to rectify this malocclusion using a number of different treatment approaches, and one of those alternatives is to distalize the maxillary posterior teeth without using extraction procedures. There have been reports of a decrease in airway space and alterations in the location of the hyoid bone following orthodontic extractions. Ng et al. [15] conducted a systematic review to evaluate the effects of bicuspid extractions and incisor retraction on the airway dimension, hyoid position, and breathing of adults and late adolescents. They came to the conclusion that orthodontic extractions for incisor retraction seemed to be especially likely to cause airway narrowing and posterior-inferior hyoid movement [16].

The imaging technique known as CBCT employs an X-ray beam that is bent to a high degree into the shape of a cone rather than a beam that is fan-shaped. It has been demonstrated that it is accurate and precise when measuring airway diameters. A precise and dependable 3D examination of the upper airway can be accomplished by using CBCT. This analysis can be performed on the upper airway [17].

One of the limitations of the CBCT imaging technique for airway analysis is the effect of head posture, neck flexion, and tongue position. A study was conducted to determine the influence of altered head or tongue posture on the upper airway (UA) volumes using MRI imaging. One supine CBCT and five sagittal MRI scans were obtained from 10 subjects in different head and tongue positions: (1) supine neutral head position (NHP) with the tongue in a natural resting position with the tip of the tongue in contact with the lingual aspect of the lower incisors (TRP); (2) head extension with TRP; (3) head flexion with TRP; (4) NHP with the tip of the tongue in contact with the posterior edge of the hard palate (THP); and (5) NHP with the tip of the tongue in contact with the floor of the mouth in contact with *Caruncula sublingualis*. The results revealed that the UA volumes, particularly the oropharyngeal volume, increased significantly with head extension and NHP with THP and decreased significantly with head flexion.

Altered head and tongue posture proved to affect the UA volumes, thus representing confounding variables during three-dimensional radiographic image acquisition [18].

The primary purpose of this study was to assess, in three dimensions, the changes that occurred in the dimensions of the airway following the distalization that was made by this appliance. On-demand application software was used to access the DICOM files that were generated from the CBCT scans. This allowed the placement of landmarks and reference planes on the image.

The findings of this research demonstrated that CMA had a beneficial impact on increasing not only the TVA but also the MCA (Tables 1-4, Figure 4). There was a considerable rise in the TVA, which went from 39.9 ± 2.7 mL to 44.9 ± 2.6 mL. There was also a large rise in the lowest confined area, which went from 213.5 ± 11.2 mm^2^ to 347.4 ± 14.9 mm^2^, respectively. The change in TVA and MCA between pre- and post-distalization was very significant (p ≤ 0.001) for each, with an increase of 12.4% and 62.7%, respectively.

In addition, the measures of the nasopharyngeal, oropharyngeal, and hypopharyngeal airway volumes all exhibited valuable changes. There was a substantial difference in the nasopharyngeal, oropharyngeal, and hypopharyngeal airway volumes. In comparison, the oropharyngeal airway volume rose from 26.9 ± 2.4 to 31.1 ± 2.3. On the other hand, the hypopharyngeal TVA increased from 4.5 ± 0.13 to 8.04 ± 0.23. The nasopharyngeal TAV increased from 13.1 ± 1.5 to 13.9 ± 1.1 between pre-distalization and post-distalization. However, the rise was highly statistically significant for both oropharyngeal and hypopharyngeal airway volumes (p ≤ 0.001), with 15.73% and 78.81% increases, respectively, in comparison to nasopharyngeal airway volume (p ≤ 0.003), which only had a 5.67% increase.

Recent research came to the conclusion that these alterations in the airway might be explained by the realization that the majority of class II treatment mechanics utilize the forward posture of the jaw as the primary factor in rectifying the sagittal connection. A change in the dimensions of the posterior airway is caused when the mandibular arch and teeth are moved forward, which in turn has an impact on the amount of space that is available for the tongue, influences the position of the hyoid bone, and causes a change in the dimensions of the airway. When it comes to the workings of the Carrière motion II appliance, class II elastics are what cause the effect of mandibular arch protraction. This, in turn, repositions the tongue anteriorly, which makes the airway more spacious [19].

Attia et al. [19] examined the influence of the CMA on the pharyngeal airway parameters in a sample of class II 20 post-adolescence patients aged from 14 to 30 using CBCT. This outcome agreed with our previous research and the study design is retrospective for both. However, there were some differences between the two studies; first, the age group of this study aged from 18 to 30 years as adults to exclude the factor of normal growth changes unlike that of Attia et al. starting from 14 years. Moreover, the sample size of this research is 18 while 20 in Attia et al. Finally, the On-Demand software was used for our study unlike the Anatomage software in Attia et al. that was installed on the CBCT scans; measurements both before and after the treatment were taken of the total airway capacity and the minimum cross-sectional area. For the total airway volume, there was a significant increase from 11.31 ± 3.02 ml to 15.2 ± 3.o3 ml. For the minimum constricted area, there was also a significant increase from 171.68 ± 56.68 mm^2^ to 212.78 ± 61.74 mm^2^. Atteia et al. claimed that the increase in the airway volume is due to the forward positioning of the mandibular arch [19].

On the one hand, no other research has assessed the alterations that may occur in the airway space after distalization of the maxillary dentition using the CMA. On the other hand, the CMA’s function was allocated by many other distalizing gadgets. Celikoglu et al. [20] carried out a study with the objective of comparing the skeletal and pharyngeal airway responses of the Herbst appliance to those of the skeletally anchored Forsus [18]. Both the upper and lower pharyngeal airway volumes were found to have increased, with the rise in the lower pharyngeal airway being determined to be statistically meaningful in the group receiving the skeletally anchored Forsus. Celikoglu et al. claimed that the reason for increasing the airway volume is mostly because the hyoid bone is posteriorly positioned in patients with skeletal class II malocclusion due to mandibular retrusion and forward movement of the mandible improves the position of hyoid bone and thus the pharyngeal airway deficiency and obstructive sleep apnea syndrome [20].

In the same line, C. Thereza-Bussolaro et al. (2019) performed a retrospective exploratory cohort study to investigate to what extent the class II malocclusion treatment with either IMEs or FFRD leads to changes in oropharyngeal airway dimensions. The results showed that both orthodontic treatment approaches appear to be associated with a similar increase in oropharyngeal airway dimensions as both techniques allowed better accommodation of the tongue and pushed downward the hyoid bone, which affects the mandibular posture [21].

A recent systematic review and meta-analysis have discussed the effects of CMA on patients with class II malocclusion on different variants like skeletal changes, dentoalveolar changes, soft tissue changes, airway changes, condylar position changes, and electromyographic activity. Regarding the airway, the review by Barakat et al. stated that only one study evaluated the changes in the airway after using CMA, which resulted in positive effects on the airway volume due to the anterior positioning of the tongue [22].

On the other hand, Park et al. found that distalization of the maxillary teeth was associated with a considerable reduction in both the oropharyngeal airway and the MCA. It is possible that this can be explained by the varied kinds of appliances that were used. In addition, there was no force that was delivered in the opposite direction between the maxillary and mandibular arches. A modified C-palatal plate, also known as an MCPP, was employed on 33 adult patients, who were then separated into groups for extraction and non-extraction. The level of distalization demonstrated a substantial inverse moderate connection with the changes in TAV and MCA when looking at the extraction group. This shows that a greater degree of distalization would result in a reduction of the airway volume to some extent. On the other hand, this was not discovered in the group that did not extract [23].

However, Chou et al. conducted a study to examine the long-term skeletodental effects, the volume of maxillary tuberosity, and changes in the airway space after maxillary molar distalization using MCPP in adolescents with class II malocclusion. The participants in the study had a malocclusion classified as Class II. After distalization, the results showed that there was a non-significant increase in the total airway volume and an increase in the airway MCA of 1.40 mm^3^ and 7.54 mm^2^, respectively. Post- distalization, the results showed that there was a non-significant increase in the TAV and MCA of 1.40 mm^3^ and 7.54 mm^2^, respectively. In addition, there were hardly any discernible differences in airway space following the conclusion of the long-term observation period [24].

Based on the information presented above, it can be concluded that the CMA was successful in producing an increase of equivalent magnitude in both TAV and MCA. The oropharyngeal airway is where most of the increase can be found. This could be explained by the fact that the appliance makes use of a reciprocal force between the maxillary arch and the mandibular arch, in terms of class II elastics, to distalize the former and create mesial movement in the latter. Because of this, the gap between the tongue and the soft palate will expand, which will in turn modify the position of the hyoid bone, and as a consequence of this, the dimensions of the posterior airway will shift [19,20].

## Conclusions

Class II malocclusion is associated with lower pharyngeal airway space volume measurement changes, according to research. This malocclusion can be corrected by distalizing the maxilla. When compared to the nasopharyngeal airways, the hypopharyngeal and oropharyngeal airways have the greatest volume gain. The CMA clearly increased the upper TAV and MCA as a primary outcome of this study. Nocturnal respiratory problems like snoring and OSA syndrome can be avoided by using the CMA, which has demonstrated positive effects on upper airway diameters through mandibular and maxillary reorientation, and soft palate repositioning, which is considered a secondary outcome of this study.

Accordingly, we present the following recommendations: 1) expand the sample size and include another appliance to compare the effect of the Carrière Motion with other appliances; 2) long-term observation of the airway changes could be helpful; and 3) evaluation of the position of the hyoid bone pre- and post-distalization. Nonetheless, the following limitations are observed: age group and gender predilection.

## Appendices

Investigator application form

_____________________________________________________________________________________

Suez Canal University

Faculty of Dentistry

Research Ethics Committee (REC)

____________________________________________________________________________________

1- Name of researcher: Heba Allah Ahmed Ahmed EIGhawy

2- Name of Department: Department of Orthodontics

3- Adress of researcher: Ismailia

a- Email: 

b-  Phone number:

c-Fax number:

4- Name (s) of co-investigator): 

5- Grade of protocol:

*M.D.Sc. (//)      *Ph.D.( )     *Doctorate degree (D.D.Sc) ( )   *Other()

*Domestic( )    *Multi-Centre within Egypt ( )          *International()

6- Title of the research: Three-Dimensional Evaluation of the Airway Space after Distalization in Adults Using Carrière Motion Appliance (CBCT Study)

7- Type of the research:

*Drug trial ( ) *Surgical technique ( ) *Investigative technique ()

*Devise study (1//:1."Survey study ( ) *Blood sampling ( )*Review of old records ( )

8- Subjects of research:

* Children (<18 years):( ) *Adults(>18 years)(//)

* Vulnerable groups (yes) or(no):No

If yes, please describe:

9- Request is being made to waive(give-up) Informed consent: Yes:( )      No:(//)

If yes, please explain why.

10- The research is for the good of society:                  No:( )

11- Study design:

a- Phase type                            11:( )      m:( )

b- Randomization:                                    No:( )

c- Placebo:                                         

d- Genetic sampling:                                

Other:           Yes:(//)     

12- Facilities for the research are available:                   

13- List the risks of the study: Exposure to Xrays/ineffective treatment

14- List the potential benefits, If any, to the subjects: The study will help the patients who suffer from class ll malocclusion to be treated without extraction and with minimum patient compliance.

15- Are the risks reasonable to the potential benefits to the subjects, if any, or to the

knowledge to be gained?                           

16- Privacy and confidentiality of subjects are assured    Yes:(//)      

17- The subject of the research could quit at any time without penalty or loss of any benefits to

which they would otherwise be entitled               

Signature of the principal investigator                    Date:

___________________________________________________________________________________

Medical history

PATIENT NAME                                                         

Patient's General Health:       (    ) Excellent      (   ) Good         (   ) Fair        (   ) Poor

Name and Address of Physician: ......................................................................................................................................

...............................................................................................................................................................................................

...............................................................................................................................................................................................

...............................................................................................................................................................................................

Is the patient currently being treated by a Physician?                                              Y or N Specify if Yes                     

Is the patient currently taking any medications?                                                       Y or N Specify if Yes                        

Is the patient allergic to any medications or substances?                                        Y or N Specify if Yes                   

Has the patient ever had an unusual reaction to analgesics (aspirin, Advil, etc.)?   Y or N Specify if Yes

Is the patient prone to prolonged bleeding (i.e. following extractions)                   Y or N

Is the patient pregnant or is pregnancy suspected?                                                Y or N

Does the patient have frequent headaches?                                                            Y or N

Does the patient have pain or clicking in jaw joints?                                                 Y or N

**Table 7:** Medical history

	Yes	No		Yes	No
Heart disease			Kidney problems		
Rheumatic fever			Venereal disease		
Heart murmur			AIDS		
Prolapsed mitral valve			Hepatitis		
Congenital heart lesions			Jaundice or liver disease		
High blood pressure			Do you currently have a dentist?		
Low blood pressure			Inflammatory rheumatism		
Artificial heart valve			Sinus trouble		
Premedication needed for dental work			Persistent cough		
Organ transplant			Arthritis		
Joint replacement			Stroke		
Diabetes			Anxiety or emotional problems		
Ulcers			Glaucoma		
Endocrine problems			Herpes (cold sores, fever blisters)		
Lung disease			Hives, skin rash		
Epilepsy			Fainting spells or seizures		
Anemia			Cancer		
Hearing disorder			Asthma or hay fever		
Latex sensitivity			Metal sensitivity		

Is there any other health information that we should know about? Y or N Specify if Yes

Signature of person completing form     Relationship to patient                 Date                                                                           

## References

[1] Khlef HN, Hajeer MY, Ajaj MA, Heshmeh O (2019). En-masse retraction of upper anterior teeth in adult patients with maxillary or bimaxillary dentoalveolar protrusion: a systematic review and meta-analysis. J Contemp Dent Pract.

[2] Bondemark L, Karlsson I (2005). Extraoral vs intraoral appliance for distal movement of maxillary first molars: a randomized controlled trial. Angle Orthod.

[3] Choi NC, Park YC, Lee HA, Lee KJ (2007). Treatment of class II protrusion with severe crowding using indirect miniscrew anchorage. Angle Orthod.

[4] Singhal A, Garg R (2013). Molar distalization by intraoral appliance. J Clin Diagn Res.

[5] (2022). Art and architecture inspire a 2021 Townie Choice Award recipient: Carriere Motion 3D Appliance for sagittal correction. https://carrieresystem.com/wp-content/uploads/2022/08/May22-ORTHOTOWN_Dr.Carriere_Motion-3D.pdf.

[6] Sandifer C, English J, Colville C, Gallerano R, Akyalcin S (2014). Treatment effects of the Carrière distalizer using lingual arch and full fixed appliances. J World Fed Orthod.

[7] Kim-Berman H, McNamara JA Jr, Lints JP, McMullen C, Franchi L (2019). Treatment effects of the Carriere(®) Motion 3D™ appliance for the correction of Class II malocclusion in adolescents. Angle Orthod.

[8] Cho HN, Yoon HJ, Park JH, Park YG, Kim SJ (2021). Effect of extraction treatment on upper airway dimensions in patients with bimaxillary skeletal protrusion relative to their vertical skeletal pattern. Korean J Orthod.

[9] Vejwarakul W, Ko EW, Lin CH (2023). Evaluation of pharyngeal airway space after orthodontic extraction treatment in class II malocclusion integrating with the subjective sleep quality assessment. Sci Rep.

[10] Hajeer MY, Millett DT, Ayoub AF, Siebert JP (2004). Applications of 3D imaging in orthodontics: part I. J Orthod.

[11] Faul F, Erdfelder E, Buchner A, Lang AG (2009). Statistical power analyses using G*Power 3.1: tests for correlation and regression analyses. Behav Res Methods.

[12] George D, Mallery P (2019). IBM SPSS statistics 26 step by step: a simple guide and reference.

[13] Ceylan I, Oktay H (1995). A study on the pharyngeal size in different skeletal patterns. Am J Orthod Dentofacial Orthop.

[14] Bourzgui F, Elmoutawakil A, Diouny S, Elquars F The impact of malocclusion on oral health related quality of life in orthodontic patients. Int J Dent.

[15] Ng JH, Song YL, Yap AU (2019). Effects of bicuspid extractions and incisor retraction on upper airway of Asian adults and late adolescents: a systematic review. J Oral Rehabil.

[16] Abdelkarim A (2012). A cone beam CT evaluation of oropharyngeal airway space and its relationship to mandibular position and dentocraniofacial morphology. J World Fed Orthod.

[17] Hakan El, Palomo J (2010). Measuring the airway in 3 dimensions: a reliability and accuracy study. Am J Orthod Dentofacial Orthop.

[18] Gurani SF, Cattaneo PM, Rafaelsen SR, Pedersen MR, Thorn JJ, Pinholt EM (2020). The effect of altered head and tongue posture on upper airway volume based on a validated upper airway analysis-an MRI pilot study. Orthod Craniofac Res.

[19] Attia K, Aboulfotouh M, Fouda A (2019). Three dimensional computed tomography evaluation of airway changes after treatment with Carriere Motion 3D Class II appliance. J Dent Maxillofacial Res.

[20] Celikoglu M, Ucar FI, Buyuk SK, Celik S, Sekerci AE, Akin M (2016). Evaluation of the mandibular volume and correlating variables in patients affected by unilateral and bilateral cleft lip and palate: a cone-beam computed tomography study. Clin Oral Investig.

[21] Thereza-Bussolaro C, Oh HS, Lagravère M, Flores-Mir C (2019). Pharyngeal dimensional changes in class II malocclusion treatment when using Forsus® or intermaxillary elastics - an exploratory study. Int Orthod.

[22] Barakat D, Bakdach WM, Youssef M (2021). Treatment effects of Carriere Motion Appliance on patients with class II malocclusion: a systematic review and meta-analysis. Int Orthod.

[23] Park JH, Kim S, Lee YJ, Bayome M, Kook YA, Hong M, Kim Y (2018). Three-dimensional evaluation of maxillary dentoalveolar changes and airway space after distalization in adults. Angle Orthod.

[24] Chou AHK, Park JH, Shoaib AM, Lee N-K, Lim HJ, Abdulwhab AA (2021). Total maxillary arch distalization with modified C-palatal plates in adolescents: a long-term study using cone-beam computed tomography. Am J Orthod Dentofacial Orthop.

